# Integrated clinicopathological model versus TNM for predicting survival in resected hepatocellular carcinoma: a retrospective cohort study

**DOI:** 10.1186/s12876-026-04895-2

**Published:** 2026-05-07

**Authors:** Gia Anh Pham, Trung Nghia Bui, Tien Cong Bui, Manh Thau Cao, Hong Son Trinh, Thi Huyen Trang Vo, Thanh Tung Pham, Thi Thu Hang Nguyen, Thanh Lam Phan, Thu Hang Nong, Thi Ngoc Tran

**Affiliations:** Department of Oncology and Radiotherapy, Viet Duc University Hospital, 40 Trang Thi Street, Hoan Kiem District, Hanoi, Vietnam

**Keywords:** Hepatocellular carcinoma, Hepatectomy, TNM staging, Microvascular invasion, ALBI grade, Prognosis, Surgical resection

## Abstract

**Objective:**

To explore prognostic factors of postoperative hepatocellular carcinoma (HCC) and compare the prognostic performance of the tumor–node–metastasis (TNM) system with an integrated clinicopathological model.

**Methods:**

We conducted a cohort study evaluating 249 patients with HCC who underwent radical liver resection between January 2015 and December 2024. Overall survival (OS) was the primary endpoint. Independent prognostic factors were determined using the Cox proportional hazards model. Model performance was assessed using the correlation index (C-index) with bootstrap internal validation. Time-dependent receiver operating characteristic (ROC) curves, decision curve analysis (DCA), and calibration plots were used to assess discrimination, clinical utility, and agreement between predicted and observed outcomes.

**Results:**

In multivariable analysis, TNM stage, microvascular invasion (MVI), resection margin status, and albumin–bilirubin (ALBI) grade were independent predictors of OS. For recurrence-free survival (RFS), independent predictors included hepatitis status, ALBI grade, TNM stage, and MVI. The integrated model demonstrated improved discrimination compared with TNM alone, with a higher optimism-corrected C-index (0.676 vs. 0.647). Time-dependent ROC analysis showed significantly higher AUC values for the integrated model at 1 year (0.725 vs. 0.669, *p* = 0.046), 3 years (0.703 vs. 0.657, *p* = 0.024), and 5 years (0.684 vs. 0.649, *p* = 0.031). DCA indicated greater net benefit across clinically relevant thresholds, and calibration plots showed good agreement between predicted and observed survival probabilities.

**Conclusion:**

An integrated clinicopathological model incorporating TNM stage, MVI, ALBI grade, and resection margin status improves prognostic performance compared with TNM alone in patients undergoing curative resection for HCC. This model may support more accurate postoperative risk stratification.

**Supplementary Information:**

The online version contains supplementary material available at 10.1186/s12876-026-04895-2.

## Background

Hepatocellular carcinoma is the most common primary form of liver cancer and one of the leading causes of cancer death globally [1, 2]. Surgical resection remains a potentially curative treatment in appropriately selected patients, where the tumor is resectable, and liver function is preserved. However, long-term survival outcomes after surgery vary considerably among patients [3, 4]. Therefore, accurate postoperative prognosis assessment is crucial in guiding follow-up strategies and supporting clinical decisions. The TNM staging system is now widely used in prognostic assessment and has been integrated into many international guidelines [5, 6]. However, because it primarily reflects anatomical extent and lacks tumor biological features and liver function, its ability to predict long-term postoperative survival remains limited [7, 8]. To overcome these limitations, an increasing number of studies are focusing on the role of histopathological features and liver function. MVI has been identified as one of the most important independent prognostic factors, closely associated with the risk of early recurrence and reduced postoperative survival time in HCC patients [9–12]. The resection margin is also significant in oncological outcomes, with tight or positive resection margins – especially in the context of concomitant MVI – indicating a more unfavorable prognosis [11, 13, 14]. In addition, the ALBI grade has been determined as an independent prognostic factor for long-term survival and for the risk of early recurrence following liver resection [15, 16]. Compared to the Child–Pugh classification, ALBI provides a more objective assessment of liver function by eliminating subjective variables such as the degree of ascites and clinical hepatic encephalopathy, thereby contributing to improved risk stratification and prediction of postoperative liver failure, especially in the Child–Pugh A group of patients [17–19]. 

Although these factors have been shown to have prognostic value, they have not yet been systematically integrated into the TNM model, and the potential benefits of incorporating them have not been fully exploited, especially in populations of patients undergoing radical surgery with long-term follow-up [5–7]. Therefore, we conducted this study to compare the effectiveness in predicting long-term survival between a TNM-only system and an integrated clinical-pathological model, including TNM staging, MVI status, surgical margins, and ALBI grade, in patients with radical HCC.

## Methods

### Ethical approval

This retrospective cohort study was conducted at Viet Duc University Hospital, Hanoi, Vietnam. The study protocol was approved by the Institutional Review Board of Viet Duc University Hospital (Approval No. IRB-VN01.021_2026.03). Because this study was retrospective and used anonymized clinical data, the ethics committee decided that informed consent was not required. All procedures complied with the ethical standards of the institutional research committee and the principles of the Declaration of Helsinki.

### Reporting guideline

This study was conducted and reported in accordance with the Strengthening the Reporting of Observational Studies in Epidemiology (STROBE) guideline.

### Study design and patients

**Fig. 1:**
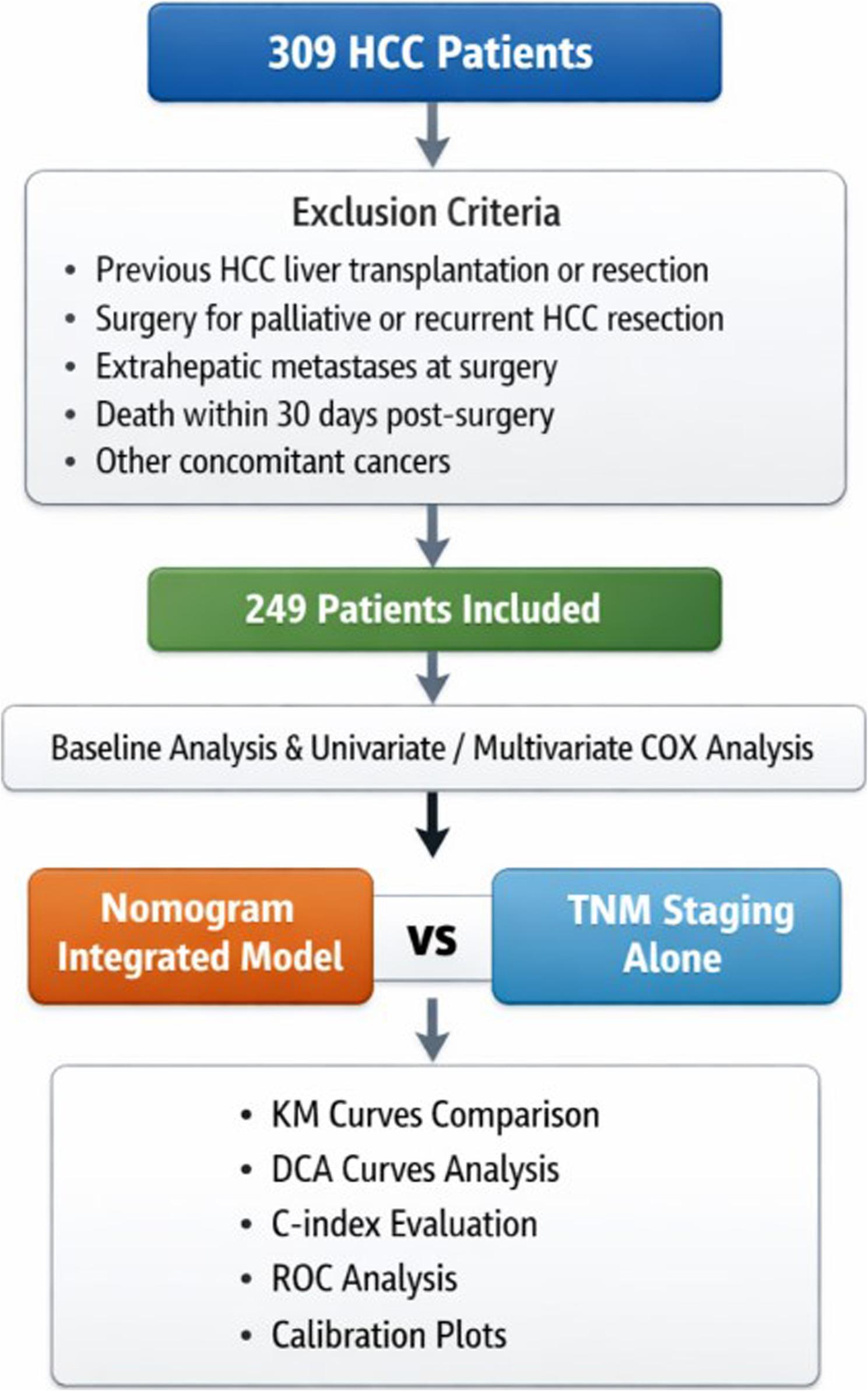
Study flowchart of patient selection

We retrospectively included 309 patients with HCC who underwent curative liver resection between January 2015 and December 2024. All eligible patients during the study period were consecutively enrolled to minimize selection bias. No patients were lost to follow-up during the study period, and complete follow-up data were available for all included patients. After applying the exclusion criteria, 249 patients were included in the final analysis. Inclusion criteria were: (1) ≥ 18 years old; (2) curative hepatectomy, (3) histopathologically confirmed HCC, and (4) complete clinicopathological and follow-up data. Exclusion criteria included: (1) prior liver resection or transplantation for HCC, (2) palliative surgery or resection for recurrent HCC, (3) presence of extrahepatic metastasis at the time of surgery, (4) death within 30 days after surgery, and (5) concomitant malignancies. The patient selection process is shown in Fig. 1.

### Data collection

Demographic, clinical, laboratory, imaging, operative, and histopathological data were derived from the hospital’s electronic medical records. Follow-up information was obtained from outpatient records and telephone interviews.

### Variables and definitions

Tumor staging was determined according to the American Joint Committee on Cancer (AJCC, 8th edition) staging system and the Barcelona Clinic Liver Cancer (BCLC) classification [20, 21]. For statistical analysis, TNM stages were grouped into early stage (I–II) and advanced stage (III–IV). MVI was defined as the presence of tumor cells within microvessels lined by endothelial cells on histopathological examination. Resection margin status was classified as R0 (negative) or R1 (positive) [4]. Liver function was evaluated using the ALBI score, calculated as: ALBI = (log₁₀ bilirubin (µmol/L) × 0.66) + (albumin (g/L) × −0.085), and categorized into grades 1–3. Major hepatectomy was defined as the removal of three or more liver segments. Satellite nodules were defined as small tumor foci adjacent to the main lesion.

### Outcome definition and follow-up

The main outcome was OS, which was defined as the time from surgery to death from any cause or last follow-up. RFS was a secondary endpoint, defined as the time from surgery to first documented recurrence or death from any cause. Patients were followed every 3–6 months during the first 2 years and every 6–12 months after, according to international guidelines [22, 23]. Follow-up continued until death or the last clinical contact. Survival status and recurrence events were obtained from medical records and the institutional follow-up system.

### Sample size considerations

We used a convenience sampling approach for this retrospective study. Therefore, a predefined sample size calculation was not conducted.

### Statistical analysis

OS and RFS were determined using Kaplan–Meier survival analysis, and group differences were calculated using the log-rank test. To determine prognostic factors, Cox proportional hazards models were applied, and hazard ratios (HR) with 95% confidence intervals (CI) were calculated using univariable and multivariable analyses. Variables showing a *p*-value < 0.20 in the univariable analysis, as well as those with clinical importance, were entered into the multivariable model. A two-sided *p*-value < 0.05 was considered statistically significant.

Collinearity diagnostics were performed using the variance inflation factor (VIF) for all variables included in the final multivariable model (body mass index, performance status, ALBI grade, TNM stage, satellite nodules, microvascular invasion, and resection margin status). All VIF values ranged from 1.008 to 1.311, indicating no evidence of significant multicollinearity.

Model discrimination was assessed using Harrell’s concordance index (C-index). Internal validation was conducted through bootstrap resampling with 1,000 iterations to derive optimism-corrected performance estimates. ROC analyses were performed at 1-, 3-, and 5-year time points, and the corresponding area under the curve (AUC) values were compared across models.

DCA was conducted to assess clinical utility across a range of threshold probabilities. Calibration was evaluated using calibration plots comparing predicted and observed survival probabilities.

All statistical analyses were performed using R version 4.4.2 and SPSS version 22.0.

### Data availability

The datasets used and analyzed in this study are not publicly available because of institutional regulations and patient privacy, but are available from the corresponding author on reasonable request.

## Results

### Clinical characteristics of the patients

A total of 249 patients who underwent curative resection for hepatocellular carcinoma were included. The median age was 57 years (range, 18–85), and 201 patients (80.7%) were male. Hepatitis B virus infection was the predominant underlying cause (66.3%). Most patients had preserved liver function, with 95.2% classified as Child–Pugh class A and 55.8% as ALBI grade 1. The median tumor size was 5.1 cm (range, 0.6–20.0), and 50.2% of tumors were ≥ 5 cm. Most patients had a single tumor (77.9%). Microvascular invasion was present in 43.8% of cases, and satellite nodules in 20.5%. Nearly half of the cohort was classified as TNM stage I (49.4%). Major hepatectomy was performed in 60.6% of patients, and R0 resection was achieved in 92.0%. Detailed baseline characteristics are shown in Table 1.

**Table 1:** Baseline characteristics of the study population

Variable	Value
Number of patients	249
Age, years	57 (18–85)
Age ≥ 60 years	104 (41.8%)
Sex	
Male	201 (80.7%)
Female	48 (19.3%)
Body mass index, kg/m²	21.5 (15.8–32.0)
BMI ≥ 23 kg/m²	177 (71.1%)
Etiology of liver disease	
Hepatitis B virus	165 (66.3%)
Hepatitis C virus	10 (4.0%)
Alcohol/NASH	27 (10.8%)
Performance status (ECOG)	
0	190 (76.3%)
≥ 1	59 (23.7%)
ALBI grade	
Grade 1	139 (55.8%)
Grade 2	110 (44.2%)
AFP, ng/mL	82.1 (1.0–179,000)
AFP ≥ 400 ng/mL	84 (33.7%)
Extent of hepatectomy	
Minor	98 (39.4%)
Major	151 (60.6%)
Portal vein thrombosis	29 (11.6%)
Tumor size, cm	5.1 (0.6–20.0)
Tumor size ≥ 5 cm	125 (50.2%)
Number of tumors	
Single	194 (77.9%)
Multiple	55 (22.1%)
TNM stage (AJCC 8th)	
Stage I	123 (49.4%)
Stage II	91 (36.5%)
Stage III	32 (12.9%)
Stage IV	3 (1.2%)
Satellite nodules	51 (20.5%)
Microvascular invasion	109 (43.8%)
Tumor differentiation	
Grade 1–2	114 (45.8%)
Grade 3–4	135 (54.2%)
Liver fibrosis stage	
F0–2	190 (76.3%)
F3–4	59 (23.7%)
Resection margin	
R0	229 (92.0%)
R1	20 (8.0%)
BCLC stage	
0	10 (4.0%)
A	114 (45.8%)
B	91 (36.5%)
C	34 (13.7%)

*Abbreviations*: Data are presented as median (range) or number (%). *ALBI* albumin–bilirubin, *AFP* alpha-fetoprotein, *BCLC* Barcelona Clinic Liver Cancer, *TNM* tumor-node-metastasis, *ECOG* Eastern Cooperative Oncology Group, *NASH* non-alcoholic steatohepatitis

### Prognostic factors for OS

Univariable and multivariable Cox regression analyses are presented in Table 2. In multivariable analysis, TNM stage (HR 2.30, 95% CI 1.28–4.13; *p* = 0.006), MVI (HR 2.17, 95% CI 1.43–3.30; *p* < 0.001), ALBI grade (HR 1.93, 95% CI 1.27–2.94; *p* = 0.002), and resection margin status (HR 2.24, 95% CI 1.14–4.40; *p* = 0.020) were identified as independent predictors of overall survival.

**Table 2:** Univariable and multivariable Cox regression analyses for overall survival

Variable	Univariable HR	95% CI	*p*-value	Multivariable HR	95% CI	*p*-value
Age (years)						
≥ 60 vs. < 60	1.09	0.72–1.64	0.697	—	—	—
Sex						
Male vs. Female	1.22	0.72–2.06	0.462	—	—	—
Body mass index (kg/m²)						
≥ 23 vs. < 23	0.86	0.73–1.06	0.049	0.85	0.74–1.12	0.078
History of viral hepatitis						
Yes vs. No	1.19	0.76–1.85	0.455	—	—	—
Alcohol consumption						
Yes vs. No	1.02	0.51–2.04	0.950	—	—	—
Performance status						
> 0 vs. 0	1.47	0.94–2.30	0.095	1.51	0.94–2.42	0.088
ALBI grade						
Grade 2–3 vs. Grade 1	1.97	1.31–2.98	0.001	1.93	1.27–2.94	0.002
Preoperative AFP (ng/mL)						
> 400 vs. ≤ 400	1.30	0.86–1.96	0.214	—	—	—
Extent of resection						
Major vs. Minor	1.20	0.78–1.85	0.405	—	—	—
Tumor size (cm)						
≥ 5 vs. < 5	1.86	1.23–2.83	0.003	—	—	—
Tumor number						
Multiple vs. Single	1.53	1.20–1.96	0.001	—	—	—
Macrovascular invasion (PVTT)						
Present vs. Absent	2.32	1.37–3.94	0.002	—	—	—
TNM stage (AJCC 8th)						
III–IV vs. I–II	2.71	1.68–4.39	< 0.001	2.30	1.28–4.13	0.006
Satellite nodules						
Present vs. Absent	1.86	1.17–2.95	0.008	1.23	0.69–2.19	0.488
MVI status						
Present vs. Absent	2.26	1.49–3.42	< 0.001	2.17	1.43–3.30	< 0.001
Resection margin status						
R1 vs. R0	1.60	0.83–3.10	0.151	2.24	1.14–4.40	0.020
Tumor differentiation						
Grade 3–4 vs. 1–2	1.17	0.78–1.76	0.451	—	—	—
Liver fibrosis (METAVIR)						
F3-4 vs. F0–2	0.92	0.46–1.84	0.822	—	—	—

Because there were few patients with stage IV disease, TNM stage (AJCC 8th edition) was grouped into early stage (I–II) and advanced stage (III–IV) for the analysis. Tumor-related variables such as tumor size and tumor number were not included in the multivariable model, as they are intrinsically incorporated into the TNM staging system, in order to avoid structural multicollinearity. A two-sided p-value < 0.05 was considered statistically significant. Liver fibrosis stages were followed by the METAVIR system [24]

*Abbreviations*: *HR* hazard ratio, *CI* confidence interval, *AFP* alpha-fetoprotein, *ALBI* albumin-bilirubin, *TNM* tumor-node-metastasis, *MVI* microvascular invasion

### OS results according to MVI status, margin resection, and ALBI grade

**Fig. 2:**
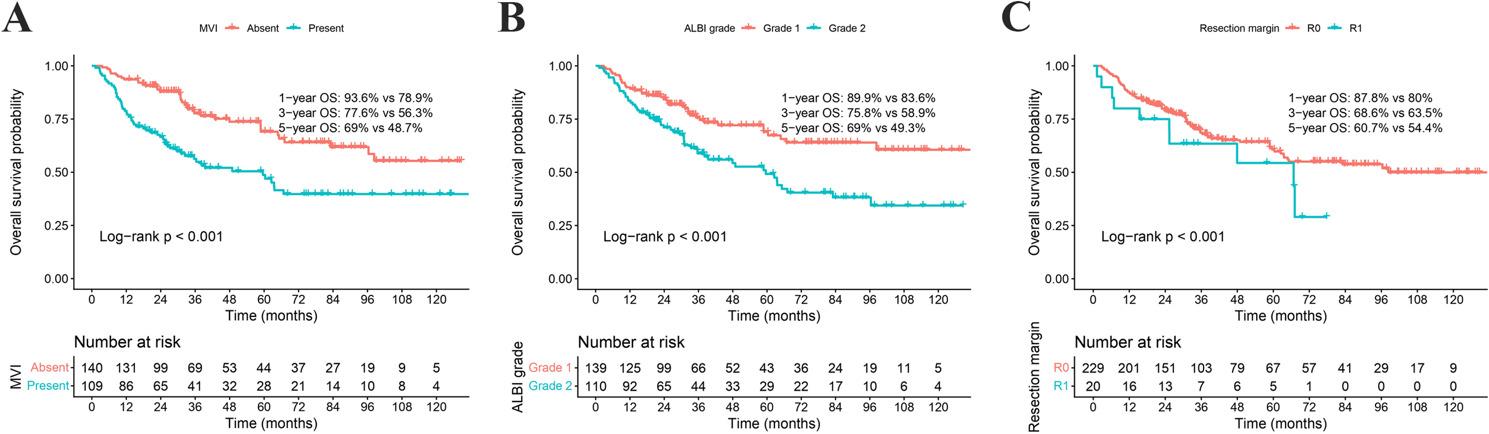
Kaplan–Meier curves for overall survival stratified by (**A**) microvascular invasion, (**B**) ALBI grade, and (**C**) resection margin status

During a median follow-up of 57.8 months (range, 1–123), the median overall survival was 98.3 months. The estimated OS rates at 1, 3, and 5 years were 86.7%, 67.6%, and 59.2%, respectively. Kaplan–Meier analysis demonstrated significantly worse survival in patients with MVI, higher ALBI grade, and positive resection margins (all *p* < 0.001; Fig. 2).

### Recurrence-free survival analysis

During a median follow-up period of 37.1 months (range, 1–128), the median RFS for the entire cohort was 55.5 months. The estimated RFS rates at 1, 3, and 5 years were 68.7%, 56.1%, and 44.2%, respectively.

Univariable Cox regression analysis (Supplementary Table S1) was performed to identify potential predictors of RFS. In the subsequent multivariable model (Table 3), several factors emerged as independent prognostic indicators for poorer RFS. Specifically, positive hepatitis status (HR 1.868, 95% CI 1.209–2.887; *p* = 0.005), higher ALBI grade (HR 1.694, 95% CI 1.155–2.483; *p* = 0.007), advanced TNM stage (HR 2.883, 95% CI 1.659–5.012; *p* < 0.001), and the presence of MVI (HR 1.849, 95% CI 1.258–2.716; *p* = 0.002) were significantly associated with an increased risk of recurrence. Notably, the extent of resection (Major vs. Minor) and the presence of satellite nodules did not significantly impact RFS in this multivariable analysis (all *p* > 0.05).

**Table 3:** Multivariate Cox regression analysis for recurrence-free survival

Variable	HR	95% CI	*p*-value
Sex (Male vs. Female)	1.489	0.866–2.560	0.150
History of viral hepatitis (Yes vs. No)	1.868	1.209–2.887	0.005
ALBI grade (Grade 2–3 vs. 1)	1.694	1.155–2.483	0.007
Extent of resection (Major vs. Minor)	1.142	0.760–1.718	0.523
TNM stage (III–IV vs. I–II)	2.883	1.659–5.012	< 0.001
Satellite nodules (Present vs. Absent)	0.868	0.512–1.474	0.601
MVI status (Present vs. Absent)	1.849	1.258–2.716	0.002

Statistically significant *p*-values (*p* < 0.05) are indicated in bold

*Abbreviations*: *HR* hazard ratio, *CI* confidence interval, *ALBI* albumin-bilirubin, *TNM* tumor-node-metastasis, *MVI* microvascular invasion

### Discrimination performance integrated model vs. TNM alone

**Fig. 3:**
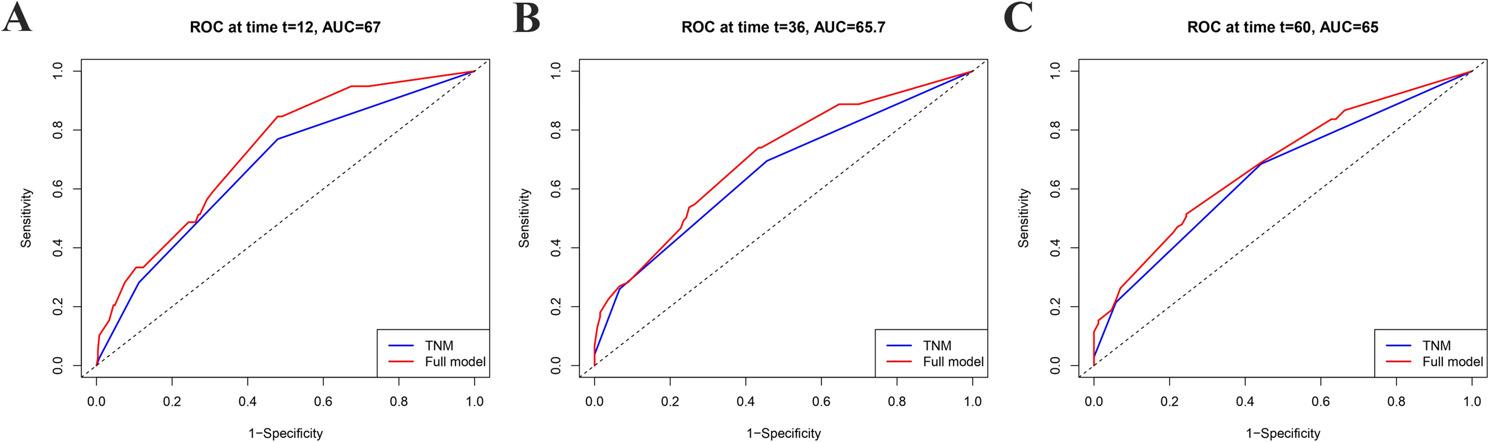
Time-dependent ROC curves comparing the TNM staging system and the integrated model at (**A**) 1 year, (**B**) 3 years, and (**C**) 5 years

**Fig. 4:**
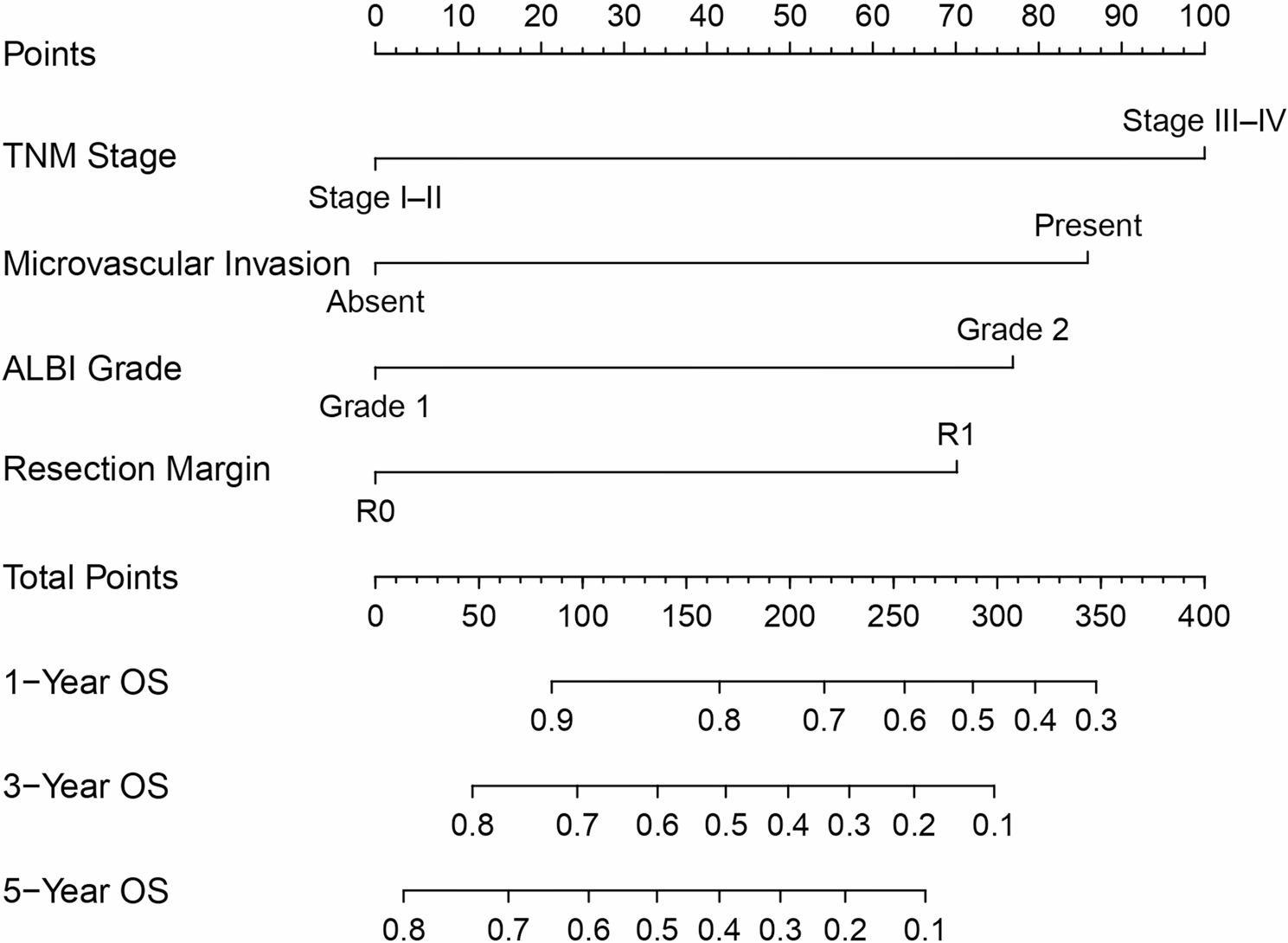
Nomogram for predicting overall survival after curative resection in patients with hepatocellular carcinoma

**Fig. 5:**
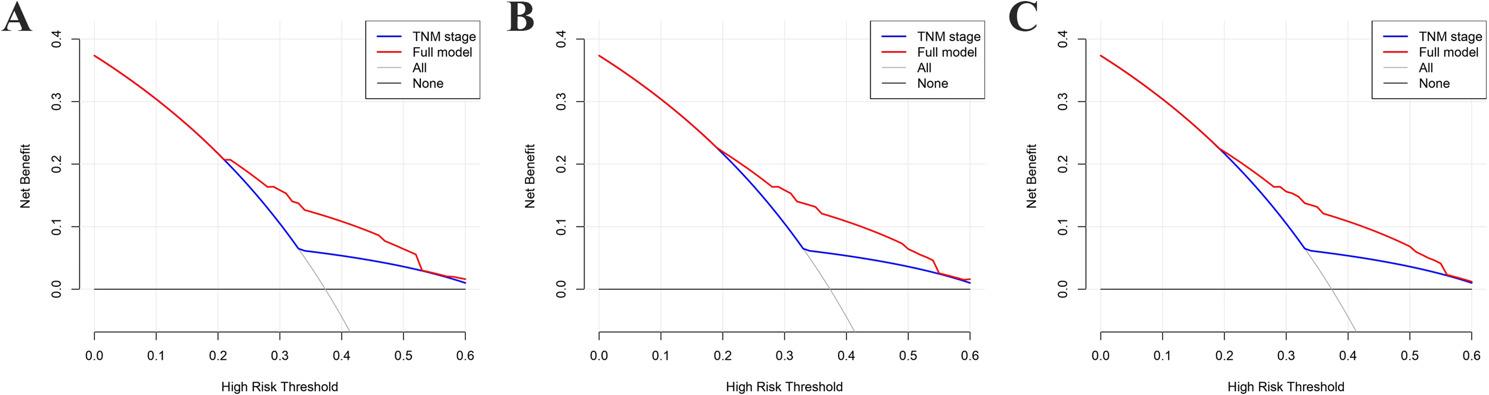
Decision curve analysis comparing clinical utility of the TNM staging system and the integrated model at (**A**) 1 year, (**B**) 3 years, and (**C**) 5 years

**Fig. 6:**
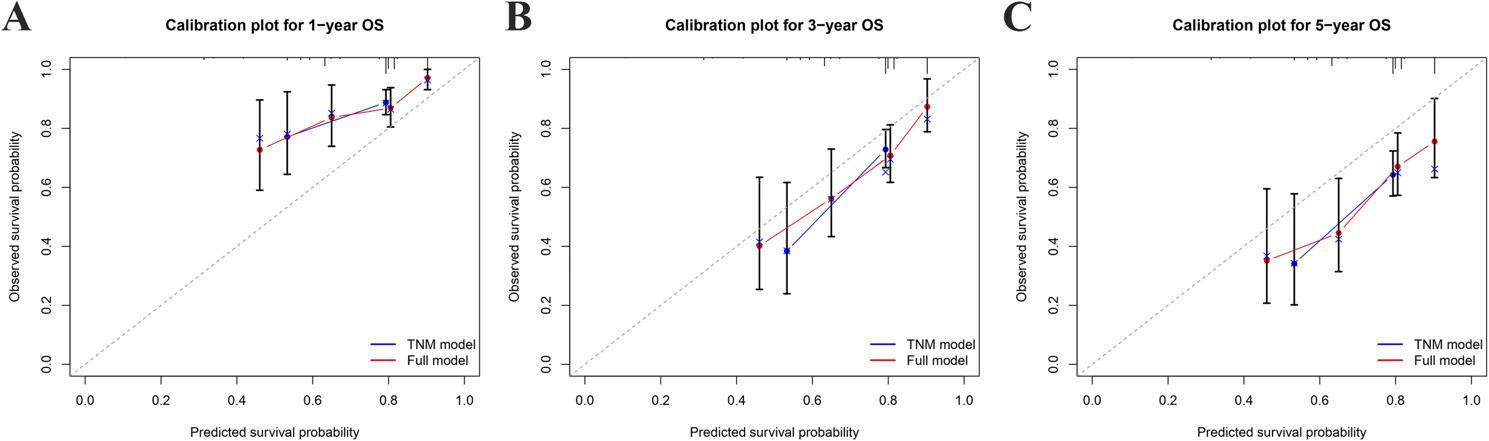
Calibration plots of predicted versus observed overall survival at (**A**) 1 year, (**B**) 3 years, and (**C**) 5 years

The optimism-corrected C-index increased from 0.647 for TNM alone to 0.676 for the integrated model (Table 4). Time-dependent ROC analysis showed significantly higher AUC values for the integrated model at 1 year (0.725 vs. 0.669, *p* = 0.046), 3 years (0.703 vs. 0.657, *p* = 0.024), and 5 years (0.684 vs. 0.649, *p* = 0.031) (Table 5; Fig. 3). Figure 4 presents the integrated nomogram model and demonstrates the estimation of individual risk based on the corresponding point scale. Decision curve analysis indicated greater net benefit of the integrated model across clinically relevant threshold probabilities (Fig. 5). Calibration plots demonstrated good agreement between predicted and observed survival probabilities at 1, 3, and 5 years (Fig. 6).

**Table 4:** C-index of prognostic models for overall survival

Model	C-index	Corrected C-index	95% CI (lower–upper)
TNM	0.724	0.647	0.652–0.788
TNM + MVI	0.722	0.656	0.657–0.781
TNM + MVI + ALBI	0.714	0.675	0.666–0.764
TNM + MVI + ALBI + Margin	0.711	0.676	0.659–0.763

**Table 5:** Comparison of time-dependent AUC between TNM and the integrated model

Time point	AUC (TNM)	AUC (Full model)	ΔAUC	95% CI	*p*-value
1 year	0.669	0.725	0.056	0.002–0.121	0.046
3 years	0.657	0.703	0.046	0.005–0.088	0.024
5 years	0.649	0.684	0.035	0.003–0.093	0.031

## Discussion

HCC remains a malignant disease with a high mortality rate and heterogeneous biological characteristics. Current staging systems, such as AJCC TNM and BCLC, are primarily based on tumor extent and liver function, which limits their ability to reflect patient survival prognosis fully [25, 26]. In addition, there is growing evidence that pathological factors such as MVI, resection margin status, along with liver function indicators such as ALBI score, play an important role in predicting long-term survival outcomes [13, 18, 27]. Previous studies have shown that a wider resection margin is associated with improved survival, especially in cases with positive MVI, while a higher ALBI grade is associated with poorer OS prognosis [28, 29]. Moreover, recent evidence has further emphasized the importance of vascular-related tumor characteristics in hepatocellular carcinoma. Tumor compression of the hepatic or portal vein has been reported as a significant predictor of MVI and the presence of satellite nodules, suggesting that macroscopic vascular interaction may reflect underlying tumor aggressiveness and dissemination potential. This highlights the biological link between vascular involvement and MVI, reinforcing the role of MVI as a key surrogate marker of tumor invasiveness and early recurrence risk [30]. Therefore, the development of integrated prognostic models that combine anatomical, tumor biology, and functional factors is crucial to improve risk stratification in an individualized manner.

Several previous studies have demonstrated that integrating factors such as ALBI, MVI, and cut-off status into prognostic models significantly improves predictive performance compared to using TNM alone. Cai et al. developed a nomogram integrating ALBI, tumor size, differentiation, and MVI, demonstrating superior discriminatory ability compared to traditional staging systems such as CLIP, BCLC, and Okuda [31]. Zhai et al. also reported an inflammation-related nomogram model integrating APRI, SIRI, and MVI indices, achieving higher AUC values ​​over time compared to the TNM AJCC version 8 at 1, 3, and 5 years [32]. In a large-scale multicenter study, Qiu et al. proposed the tumor burden–AFP–ALBI score, showing superior predictive ability of postoperative OS compared to the BCLC system [33]. In addition, a recent study by Liu et al. also noted a nomogram combining inflammatory markers, ALBI, AFP, and viral infection status that improved prognostic accuracy compared to TNM staging in patients with resectable early-stage HCC [34]. Overall, these results suggest that integrated models have the potential to improve prognostic value by simultaneously incorporating anatomical, biological, and functional factors.

The integrated model in our study, including TNM stage, MVI, ALBI grade, and resection margin, showed better prognostic performance than TNM alone. It had a higher C-index (0.676) and slightly higher AUC at 1, 3, and 5 years. Decision curve analysis also showed better clinical benefit, and calibration demonstrated good agreement between predicted outcomes and observed results. Although the improvement was modest, the model combines important factors from different aspects, including tumor stage, tumor biology, liver function, and surgical outcome. These factors provide a more complete view of the patient’s condition and may help improve risk stratification [29, 32, 34]. 

From a surgical perspective, emerging data also support that the prognostic impact of operative strategies may be mediated more by tumor biology than by technical factors alone. A recent study by Luo et al. demonstrated that resection of the middle hepatic vein, although associated with increased operative complexity and transient postoperative liver dysfunction, did not adversely affect long-term outcomes such as OS and RFS. Instead, survival was primarily determined by tumor-related factors such as BCLC stage, AFP level, and liver function (ALBI grade). These findings further support the rationale that incorporating biological and functional parameters—rather than surgical extent alone—may provide a more accurate prediction of prognosis [35]. 

Importantly, the findings from RFS analysis further supported the robustness of the proposed model. In addition to overall survival, TNM stage, MVI, ALBI grade, and hepatitis status were independently associated with recurrence risk. The consistency between OS and RFS findings suggests that the model captures both tumor aggressiveness and early recurrence biology, reinforcing its clinical relevance for postoperative risk stratification. Although RFS was additionally analyzed to support early postoperative risk stratification, OS remained the primary endpoint, and the prognostic model was therefore developed and optimized based on OS as the principal outcome, with RFS serving as a complementary supportive analysis.

Clinically, the integrated model can help identify high-risk patients after radical surgery, thereby guiding strategies for closer monitoring or consideration of adjuvant treatments. Prior studies have shown that these models can additionally stratify patients at the same anatomical stage into different risk groups with marked differences in survival [33, 34]. Conversely, relying solely on TNM may miss important information related to tumor biology and liver function [32]. Therefore, this integrated model may help support a more personalized approach to assessing risk in hepatocellular carcinoma, leading to better management and treatment strategies [25, 36]. 

This study has several advantages, including the use of clinical practice data, a relatively complete set of clinical-pathological variables, and a relatively long median follow-up time (57.8 months). The prognostic value of the model was comprehensively assessed through parameters such as discrimination, calibration, and decision curve analysis. However, our study also has some limitations. The sample size was relatively small, and the number of patients with a low R1 cut-off area somewhat limited the value of the subgroup analyses. Although the R1 cut-off area has been shown to be associated with poor long-term survival prognosis in previous studies, the current results still need to be interpreted cautiously [4, 37]. In addition, this study has some limitations. First, it is a retrospective study from a single center, which may lead to selection bias. We also could not fully control all confounding factors. Second, the data come from one hospital in Vietnam, so the results may not be the same in other regions or healthcare systems. Therefore, our findings may not be fully generalizable. Future multicenter and prospective studies are needed to confirm our results. In addition, our study only included internal validation using bootstrap resampling, and external validation was not performed due to limited data availability. Therefore, the generalizability of the model to other populations remains uncertain. Future studies with independent external cohorts are needed to confirm the reliability and applicability of this model in different clinical settings. Finally, the prognostic value of the model tended to decrease at longer time points, which is explained by changes in tumor characteristics and the influence of post-relapse treatment methods during long-term follow-up [32]. 

In summary, the integrated clinical-pathology model, including TNM staging, ALBI grade, microvascular invasion, and resection margin status, showed improved predictive ability for overall survival compared to the AJCC TNM system alone in patients with radical HCC. Although the improvement was not significant, this model still contributes to strengthening the prognostic value of multifactorial models in the present and future. Therefore, more future studies are needed, especially multicenter, prospective studies, to confirm the results and clarify the role of integrated prognostic models in clinical practice.

## Conclusion

An integrated clinicopathological model including TNM stage, MVI, ALBI grade, and resection margin showed improved prognostic performance compared with TNM alone in resected HCC. Although the improvement was modest, the model provides a more comprehensive assessment of patient prognosis. In addition, the consistency between OS and RFS findings further supports the robustness of the model in capturing both long-term outcomes and early recurrence risk. Further studies with bigger sample sizes and external validation are required to confirm that integrated models can be applied widely and used effectively in clinical practice.

## Supplementary Information

Supplementary Material 1.

Supplementary Material 2.

## Data Availability

The datasets used and analyzed in this study are not publicly available because of institutional regulations and patient privacy, but are available from the corresponding author on reasonable request.
