# Supplementary material for: Integrated clinicopathological model versus TNM for predicting survival in resected hepatocellular carcinoma: a retrospective cohort study
Source: BMC Gastroenterol. 2026 May 7;26:391. doi: 10.1186/s12876-026-04895-2 (PMC13321625; doi:10.1186/s12876-026-04895-2)
Supplement: Supplementary file 1 — Supplementary Material 1. [file 12876_2026_4895_MOESM1_ESM.docx]

**Title**: Integrated Clinicopathological Model versus TNM for Predicting Survival in Resected Hepatocellular Carcinoma: A Retrospective Cohort Study

**SUPPLEMENTARY**

**Table S1. Univariate Cox regression analysis for RFS**

| **Variable** | **HR** | **95% CI** | **p-value** |
| --- | --- | --- | --- |
| Age (≥60 vs <60) | 0.845 | 0.574–1.245 | 0.395 |
| Sex (Male vs Female) | 1.743 | 1.025–2.964 | 0.040 |
| Body mass index (≥23 vs <23) | 0.839 | 0.551–1.275 | 0.410 |
| History of viral hepatitis (Yes vs No) | 1.550 | 1.013–2.372 | 0.044 |
| Alcohol consumption (Yes vs No) | 0.663 | 0.323–1.361 | 0.263 |
| Performance status (>0 vs 0) | 1.272 | 0.836–1.935 | 0.260 |
| ALBI grade (Grade 2–3 vs 1) | 1.594 | 1.097–2.316 | 0.014 |
| Preoperative AFP (>400 vs ≤400) | 1.177 | 0.801–1.727 | 0.406 |
| Extent of resection (Major vs Minor) | 1.345 | 0.903–2.001 | 0.145 |
| Tumor size (≥5 cm vs <5 cm) | 1.551 | 1.066–2.257 | 0.022 |
| Tumor number (Multiple vs Single) | 2.040 | 1.358–3.065 | 0.001 |
| Macrovascular invasion (PVTT) | 1.850 | 1.087–3.148 | 0.023 |
| TNM stage (III–IV vs I–II) | 2.926 | 1.849–4.628 | <0.001 |
| Satellite nodules (Present vs Absent) | 1.646 | 1.064–2.547 | 0.025 |
| MVI (Present vs Absent) | 1.865 | 1.284–2.711 | 0.001 |
| Resection margin (R1 vs R0) | 1.199 | 0.606–2.373 | 0.602 |
| Tumor differentiation (G3–4 vs G1–2) | 0.920 | 0.634–1.335 | 0.661 |
| Liver fibrosis (F3–4 vs F0–2) | 0.973 | 0.534–1.771 | 0.927 |

**A
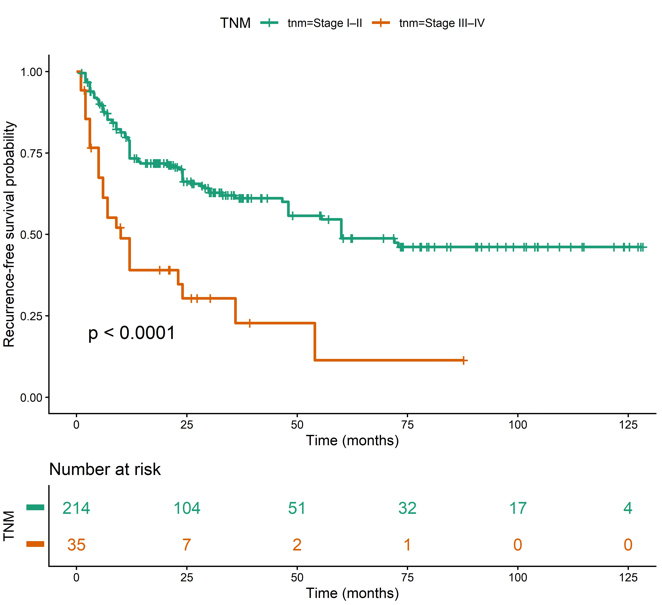
B
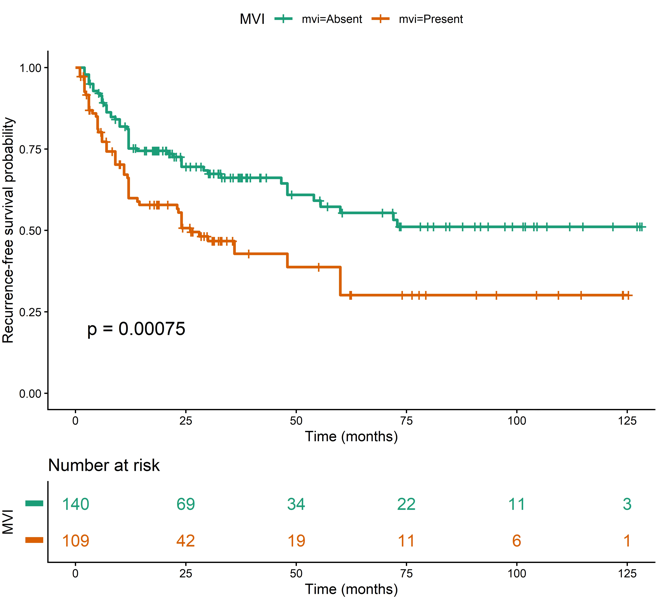
C
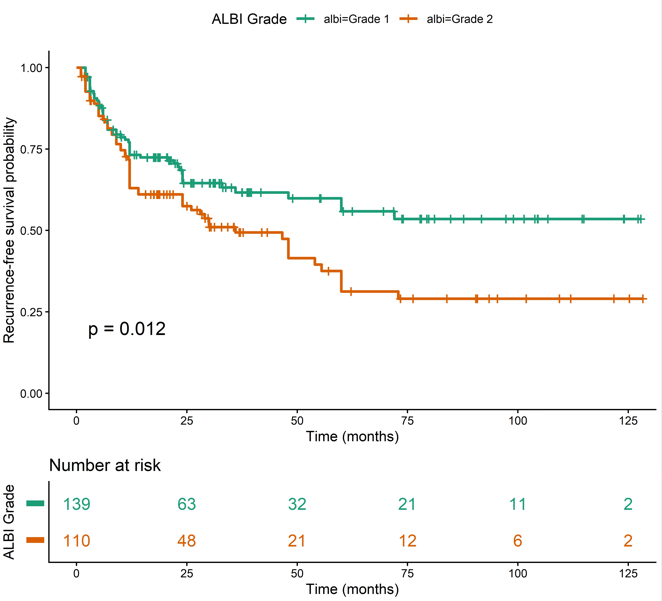
**

**Figure S1. Kaplan–Meier analysis demonstrated significantly worse RFS in patients with advanced TNM stage (A); MVI status (B); and ALBI grade (C).**
