# Supplementary figures and images for: Integrated clinicopathological model versus TNM for predicting survival in resected hepatocellular carcinoma: a retrospective cohort study
Source: BMC Gastroenterol. 2026 May 7;26:391. doi: 10.1186/s12876-026-04895-2 (PMC13321625; doi:10.1186/s12876-026-04895-2)

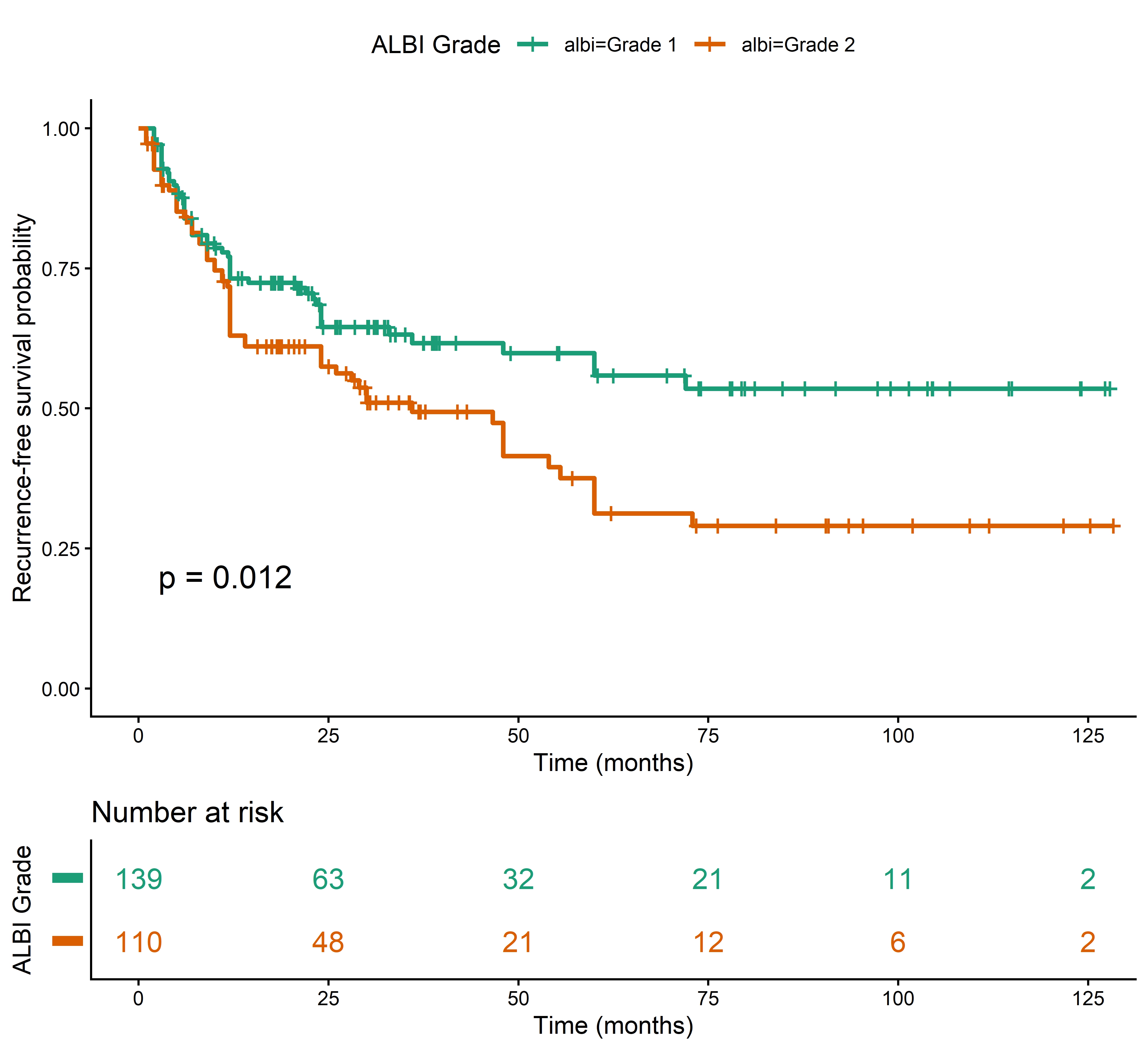

Supplement: Supplementary file 2 — Supplementary Material 2. [file 12876_2026_4895_MOESM2_ESM.zip › FigureS1C.jpg]

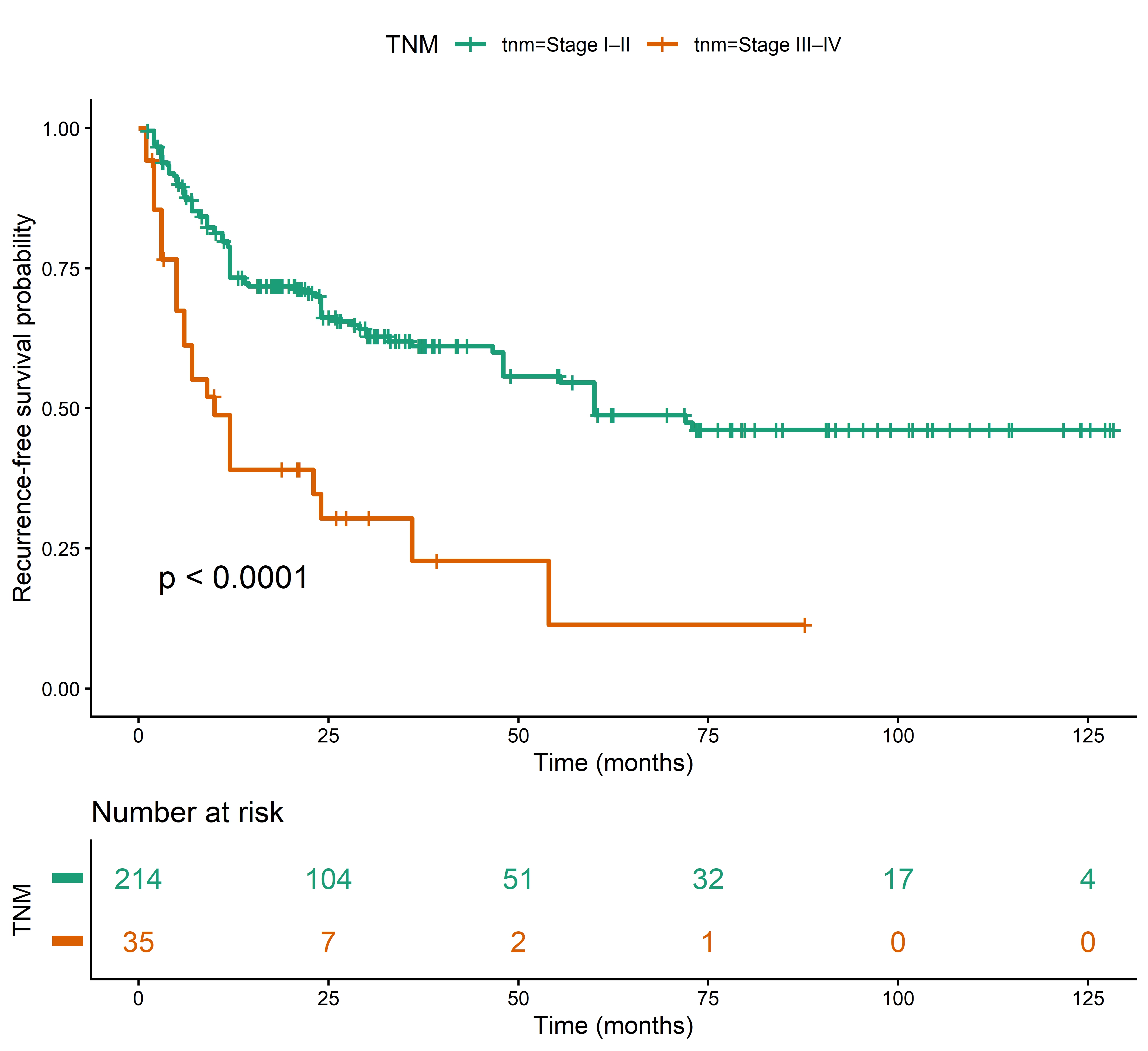

Supplement: Supplementary file 2 — Supplementary Material 2. [file 12876_2026_4895_MOESM2_ESM.zip › FigureS1A.jpg]

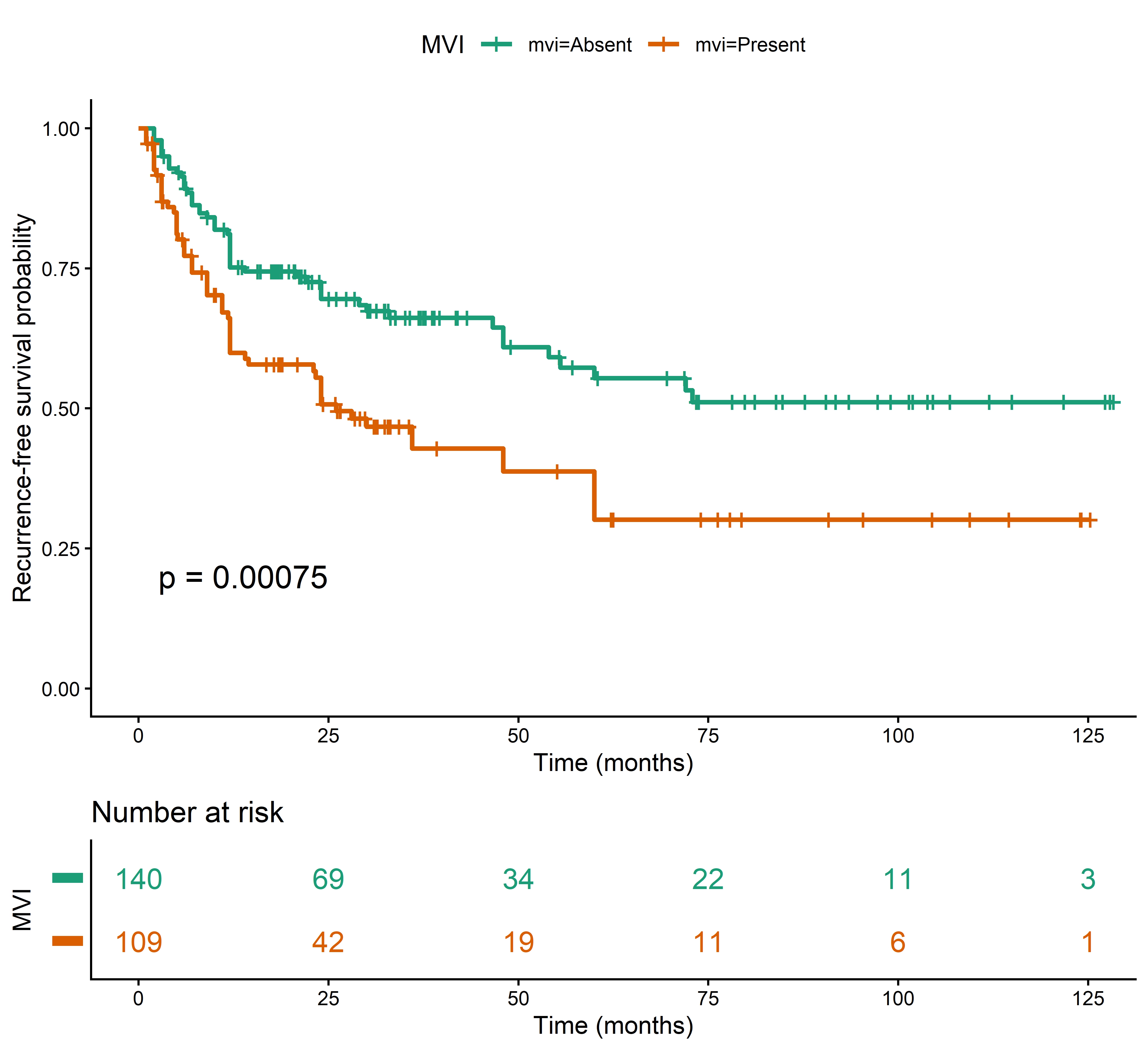

Supplement: Supplementary file 2 — Supplementary Material 2. [file 12876_2026_4895_MOESM2_ESM.zip › FigureS1B.jpg]
